# The Effects on Health Behavior and Health Outcomes of Internet-Based Asynchronous Communication Between Health Providers and Patients With a Chronic Condition: A Systematic Review

**DOI:** 10.2196/jmir.3000

**Published:** 2014-01-16

**Authors:** Catharina Carolina de Jong, Wynand JG Ros, Guus Schrijvers

**Affiliations:** ^1^Julius Center for Health Sciences and Primary CareUniversity Medical Center UtrechtUniversity of UtrechtUtrechtNetherlands; ^2^Stichting Transmurale Zorg Den Haag EOThe HagueNetherlands

**Keywords:** chronic disease, telecommunications, Internet, telemedicine, health services, delivery of health care, medical informatics, electronic mail, self-care, self-efficacy

## Abstract

**Background:**

In support of professional practice, asynchronous communication between the patient and the provider is implemented separately or in combination with Internet-based self-management interventions. This interaction occurs primarily through electronic messaging or discussion boards. There is little evidence as to whether it is a useful tool for chronically ill patients to support their self-management and increase the effectiveness of interventions.

**Objective:**

The aim of our study was to review the use and usability of patient-provider asynchronous communication for chronically ill patients and the effects of such communication on health behavior, health outcomes, and patient satisfaction.

**Methods:**

A literature search was performed using PubMed and Embase. The quality of the articles was appraised according to the National Institute for Health and Clinical Excellence (NICE) criteria. The use and usability of the asynchronous communication was analyzed by examining the frequency of use and the number of users of the interventions with asynchronous communication, as well as of separate electronic messaging. The effectiveness of asynchronous communication was analyzed by examining effects on health behavior, health outcomes, and patient satisfaction.

**Results:**

Patients’ knowledge concerning their chronic condition increased and they seemed to appreciate being able to communicate asynchronously with their providers. They not only had specific questions but also wanted to communicate about feeling ill. A decrease in visits to the physician was shown in two studies (*P*=.07, *P*=.07). Increases in self-management/self-efficacy for patients with back pain, dyspnea, and heart failure were found. Positive health outcomes were shown in 12 studies, where the clinical outcomes for diabetic patients (HbA1c level) and for asthmatic patients (forced expiratory volume [FEV]) improved. Physical symptoms improved in five studies. Five studies generated a variety of positive psychosocial outcomes.

**Conclusions:**

The effect of asynchronous communication is not shown unequivocally in these studies. Patients seem to be interested in using email. Patients are willing to participate and are taking the initiative to discuss health issues with their providers. Additional testing of the effects of asynchronous communication on self-management in chronically ill patients is needed.

## Introduction

### Background

Self-management is a central concept of health care that is increasing in popularity. This is because people strive to be autonomous and also because there is an increase in the global population, in the number of chronic diseases people have, and in the length of time people endure a chronic disease [1]. To make health care attainable for all, it is necessary to implement self-management on as large a scale as is justifiable. The level of patient participation is a key aspect in the realization of self-management. Research shows that patient participation results in improved adherence and health outcomes [2-4]. It is therefore important to understand what makes patients participate in their own health care.

The transition towards self-management is also visible in the new definition of health that experts have recommended to the World Health Organization (WHO): “the ability to adapt and self-manage in the face of social, physical, and emotional challenges” [5]. The earlier definition, which dates to 1948 [6], describes health as “a state of complete physical, mental and social well-being and not merely the absence of disease or infirmity”, which today seems unattainable. Ursum gives a clear operationalization of self-management as “the individual ability of a person to prevent health problems from arising or, if they do arise, to adapt to the symptoms, the treatment, the physical, psychological and social consequences of the health problem and adjust their lifestyle. In this way persons are able to monitor their own health and to react in a way that results in a satisfactory quality of life” [7]. Thus, self-management is an ongoing process in the lives of people, even when they are not receiving care from health care providers. It requires that all people, but certainly patients, need to have open communication with health care providers to be able to take responsibility. This definition suggests that an important focus of health care research should be evidence-based ways of improving self-management by patient participation [8].

### Chronic Patients and Modern Communication Technology

Modern information and communication technologies (ICT) provide new ways for patients to participate in their own health care. Internet interventions have been developed to record, measure, monitor, and manage the delivery of health care [9]. These interventions enable patients to remotely supply providers with personal health information and for providers to remotely deliver instructions. For instance, patients with cardiac problems can play an active role in diagnosis by monitoring and communicating their health data so that the professional can make a diagnosis [10,11]. Another option is to give patients access to specific interactive self-care techniques such as back pain management training. A third option is inviting the patient to view their electronic health record (EHR). The patient can log in to the EHR and comment on the content. Some evidence shows that interventions via the Internet also improve self-care behavior and health outcomes in patients with chronic diseases [12-16]. Especially in the case of chronically ill patients living at home, these Internet-based interventions are seen as a promising development to improve the quality and safety of health care [17]. However, robust research on the effectiveness and consequences of these interventions is needed to guide large-scale implementation [18,19].

### Specifically Asynchronous Communication

The above-mentioned Internet interventions are created according to a standard pattern based on one-way communication with not much scope for interaction. However, Internet interventions can also be combined with interactive communication tailor-made for individual patients [12,20,21]. Interactive communication can be synchronous (concurrent by telephone consultation or videoconferencing) or asynchronous (non-concurrent by, for example, email or discussion board). This latter form of digital communication has the advantage that the patient and the provider do not need to use it concurrently. For example, patients can pose a question about the organization of their care or a health concern at the moment that it worries them and do not have to wait until the next planned consultation. For health care professionals, it means they can react to patient questions at a time that is suitable for them. Asynchronous communication is not suitable in urgent situations because there is a time gap in the communication. The asynchronous options make it possible to deliver tailor-made self-management support to large numbers of patients with a chronic disease [20].

### Current Investigation

This review examines publications that describe the effect of digital asynchronous communication between chronically ill patients and health care providers. The first research question is whether this type of communication works: do patients and providers actually use this form of interactive communication and how do they evaluate the usability? The second question reviewed is whether this form of interactive communication helps: does it have an effect on health behavior, health outcomes, and patient satisfaction?

## Methods

### Definitions

Digital asynchronous communication is defined as electronically mediated communication in which the participants do not communicate concurrently. Examples of asynchronous communication in health care are electronic messaging (email) and bulletin boards. Patients with chronic conditions have one or more chronic diseases, which are defined as diseases with a long duration and generally slow progression [1].

### Literature Searching Methods

The systematic review was conducted using the PICO method [22]. The keywords (MeSH terms [Medical Subject Headings]) used were chronic disease, telecommunications OR Internet OR telemedicine OR health services OR delivery of health care OR medical informatics OR electronic mail, self-care, self-efficacy. The search was filtered for Randomized Controlled Trials (RCTs), adults, English language, and publication period of 2001-2013. The search was limited to studies conducted from 2001 onward because Internet access for individuals from their homes has increased since the turn of the century [23] and interactive asynchronous communication thus became an option for more people. The search procedure consisted of the following steps:

1. PubMed and Embase databases were searched.
2. Duplicates were removed.
3. Titles and abstracts were scanned for Internet-based interventions.
4. Full text analysis was undertaken to select studies
  - describing asynchronous communication between patient and provider, alone or as part of an intervention;
  - where patients were able to initiate communication at any time of the day (24/7);
  - directed at self-management;
  - where control groups were free of any digital intervention and received usual care.
5. The bibliographies of the articles included were manually searched to identify additional relevant articles.

### Quality Appraisal

The methodological quality of the studies was evaluated by applying the National Institute for Health and Clinical Excellence (NICE) criteria for RCTs [1,24,25]. Selection bias, performance bias, attrition bias, and detection bias were assessed.

### Research Questions

#### Do Patients and Providers Use Asynchronous Communication Within Internet-Based Interventions and Do They Find It Usable?

To answer this question, the use of asynchronous communication in digital interventions by patients was assessed by determining frequency of access; number of patients who use the digital intervention; number of messages; and usability of interventions, including asynchronous communication (this refers to experiences regarding the actual (technical) use of the ICT with a focus on ease of use, clarity, and attractiveness).

#### Does the Use of Asynchronous Communication Within Internet-Based Interventions Affect Health Behavior and Health Outcomes?

To answer this question, the present study assessed the effects of using digital interventions, including asynchronous communication, on health behavior (knowledge, health care utilization, and self-efficacy/self-management); health outcomes (clinical parameters, physical symptoms, quality of life); and patient satisfaction.

The results were reported as significant if *P*<.05.

## Results

### Characteristics of the Studies

**Figure 1:**
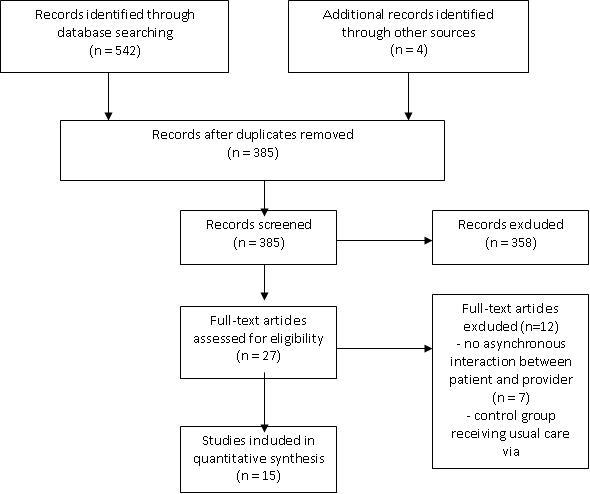
Search results.

As shown in Figure 1, the search identified 311 studies in PubMed and 231 in Embase. Four additional studies were retrieved via the bibliographies of the retrieved studies. The elimination of duplicates resulted in 385 studies. All abstracts were screened and 27 studies were identified that specifically focused on Internet-based intervention. After full text analysis, 20 studies were identified that described asynchronous digital communication between patients and providers as part of the intervention. Furthermore, five studies in which the control group received usual care via the Internet were eliminated. Ultimately, 15 studies were found to meet the inclusion criteria and were thus included in the review (Figure 1).

Table 1 shows the characteristics of the samples of the studies. The studies varied in geographic location, sample size, mean age of the sample, and nature of the chronic disease (Table 1). Eleven of the 15 studies were performed in the United States, two in Europe (Portugal, The Netherlands), one in Australia, and one in Asia (Korea). The sample sizes varied from 0-50 [26], 50-100 [9,27-29], 100-150 [30-34], and more than 150 [35-39].

In 9 studies, the mean age of participants was approximately 50 years (range 45-57) [28-31,34-36,38,39]. In four studies, the mean age was over 60 years [9,27,32,33], and in two studies, the mean age was lower (range 29-36) [26,37]. The nature of the chronic disease differed in the samples, including unspecified chronic illnesses [30,35,38,39], chronic pain [27,36], diabetes [9,29,31,32], asthma [26,37], chronic obstructive pulmonary disease [33], chronic neurological conditions [28], and congestive heart failure [34].

Table 2 shows the characteristics of the interventions of the studies. The interventions were diversely directed. Eight studies focussed on self-care techniques [27,28,30,33,35,36,38,39], six on monitoring disease and symptoms [9,26,29,31,32,37], and three on sharing an EHR [29,31,34].

An assessment was carried out to determine whether the interventions were complementary or a substitute for usual care because of the consequences for the interpretation of the results. One might evaluate interventions that are complementary to usual care as effective if the study shows improved outcomes, whereas interventions that are a substitute for usual care can be evaluated as effective if the study shows no differences compared with usual care. Most interventions were complementary (n=11), but some were a substitute for usual care (n=4).

**Table 1:** Characteristics of the sample.

Primary author	Country	Sample size^a^, n	Chronic disease	Mean age, yrs (range)	Female, %	Recruitment
Berman [27]	United States	I: 41 C: 37	Chronic pain	65.8 (55-91)	87.2	Mass media
Bond [9]	United States	I: 31 C: 31	Diabetes, type 1 and 2	67.2	45	Provider
Cruz [26]	Portugal	Cross-over I: 21	Asthma	29 (18-62)	71	Provider
Ghahari [28]	Australia	IA: 34 IB: 28 C: 33	Chronic neurological conditions: MS, Parkinson’s, post-polio	50.25 (23-90)	81.1	Mass media
Hill [30]	United States	I: 61 C: 59	Chronically ill rural women	52.2 (35-65)	100	Mass media
Kwon [31]	Korea	I: 51 C: 50	Diabetes type 2	54.1	30.9	Provider
Lin [35]	United States	I: 305 C: 301	Acad. internal medicine practice	51	51	Provider
Lorig [36]	United States	I: 296 C: 284	Chronic back pain	45.5	38.5	Mass media
McMahon [32]	United States	I: 52 C: 52	Diabetes, HbA1c ≥9	63.5	0	Provider
Meer [37]	Netherlands	I: 101 C: 99	Asthma	36.5 (19-50)	69.5	Provider
Nguyen [33]	United States	IA: 43 IB: 41 C: 41	COPD	68.7	46	Mass media
Ralston [29]	United States	I: 42 C: 41	Diabetes, type 2, HbA1c≥7	57.3	49.4	Provider
Ross [34]	United States	I: 54 C: 53	Congestive heart failure	56	77	Provider
Weinert [39]	United States	IA: 54 IB: 58 C: 64	Chronically ill rural women	51.8 (30-69)	100	Mass media
Weinert [38]	United States	I: 155 C: 154	Chronically ill rural women	55.5	100	Mass media

^a^I=intervention, C=control.

**Table 2:** Characteristics of the intervention.

Primary author	Intervention				Complement or substitute for usual care	Length, wks
	Self-care technique	Monitor disease/ symptom	Share EHR	Description		
Berman [27]	x			Online mind-body: Facilitator sends prompts and answers questions using asynchronous communication	complement	6
Ghahari [28]	x			A. Online fatigue self-management program: Facilitators logged on daily and responded to participant entries, posed questions and provided encouragement to the intervention group. B. Online information-only self-management program: In the information-only group, facilitators checked for technical problems and sent standardized weekly reminders to read the information.	complement	13
Hill [30]	x			Computer intervention on psychological status: Online peer-led support group with health teaching; included an asynchronous chat room and an email function giving access to each other and to research team.	complement	22
Lin [35]	x			Patient portal: To send secure messages directly to their physicians and to request appointments, prescription refills and referrals; also included clinical messaging.	complement	26
Nguyen [33]	x			A. Internet-based dyspnea self-management program (eDSMP): Web diary, personalized reinforcement, feedback emails, and a discussion board. B. Face-to-face fDSMP: Paper diary, personalized reinforcement, and feedback telephone calls	complement	52
Weinert [38]	x			Peer-led support group and a self-study health coaching unit	complement	24
Weinert [39]	x			A. Computer-based intervention providing online support and health information B. Health information only	complement	53
Lorig [36]	x			Closed, moderated email discussion group; book, videotape.	complement	52
Bond [9]		x		Web-based intervention with disease monitoring, coaching, motivational, and social support	complement	26
Ralston [29]		x	x	Shared electronic record and email with providers	complement	52
Ross [34]			x	Web-based online review of EHR and email messaging directed at clarifying doctor’s assessment and instructions	complement	52
Cruz [26]		x		E-diaries and asthma self-management with PIKO-1 e-tool: Immediate feedback using secure messaging	substitute	8
McMahon [32]		x		Care website with educational modules, monitoring system, and internal messaging system	substitute	52
Meer [37]		x		Internet-based self-management plus education and communication with an asthma nurse	substitute	52
Kwon [31]		x	x	Website for monitoring and communication	substitute	13

### Methodological Quality of Studies Included

In Multimedia Appendix 1, the methodological quality of the RCTs in this study is presented.

Risk for selection bias, or systematic differences between the comparison groups, existed in three studies [30,36,38]. Two studies did not describe how the participants were randomized and up to which point the investigators were blinded to allocation [30,36]. Two studies did not describe how the groups compared at baseline [30,38]. In all three studies, the participants were invited through mass media, which means that the assignment of patients to a group was partially concealed because the investigators did not know the participants [30,36,38]. The partial concealment was not considered a high risk for bias.

Risk for performance bias, or systematic differences between groups in the care provided (apart from the intervention under investigation), was low. In all studies, the comparison groups received the same care except for the intervention studied. In all 15 studies, it was clear that participants were not blinded to the treatment allocation due to the physical character of the intervention. In the studies by Ghahari [28], Nguyen [33], and Weinert [38], where two interventions (intense and less intense) were compared with usual care, patients in the intervention groups knew that they were taking part in the intervention but did not know whether they were participating in the intense or less intense intervention group.

The investigators were kept blinded in three studies. In the study by Bond [9], it was possible to keep the investigators blinded because the outcomes were measured at the beginning and the end of the intervention during a home visit. Cruz [26] used a crossover design in which all participants took part in the treatment and control group sequentially so that blinding of investigators was not an issue. In the study by Ghahari [28], the investigators were partially blind to how the outcomes were ascertained because they were involved only in one arm of the three-armed study and were blind to the survey results.

Risk of attrition bias, or systematic differences between the comparison groups with respect to dropouts, hardly existed in the studies. All groups within the studies were followed for an equal period of time. They were comparable with respect to the availability of outcome data and for treatment completion. Only Weinert [38] did not describe data on treatment completion.

Risk of detection bias, or bias in how outcomes are ascertained, diagnosed, and verified, showed more diversity. The length of follow-up was found to be on the short side in Berman (6 weeks) [27] and Cruz (8 weeks) [26]. The validity and reliability of the outcome measures was unclear in Cruz [26] because the psychometric qualities were not discussed. In Weinert [38], the quality of the outcome measures was not described. In three studies [9,28,30], the investigators were kept blind to the participants’ exposure to the intervention and to other important confounding prognostic factors.

In conclusion, a total score for the studies was derived that summed up the risk for the four categories of bias, showing that Berman [27], Hill [30], and Weinert [38] have some risk to methodological quality. The other 12 studies show low risk.

### Does It Work? Use and Usability

In Multimedia Appendix 2, the results describing the use of the intervention, health behaviour, and health outcomes are presented. Although all 15 studies studied an intervention including asynchronous communication, outcomes concerning use of the intervention, such as accessing the intervention and the use of electronic messaging, were not reported in all studies. Twelve studies described the use of the intervention by patients. In six studies, the specific use of the asynchronous communication was also subject of the study.

To use the asynchronous communication, the patients accessed a website and then logged in to a patient portal. It was then possible for them to communicate asynchronously with their providers by using email, viewing their EHR, or using a discussion board. Three studies that examined sharing EHR [29,31,34] described data about how the intervention was accessed. These patients accessed the intervention between 1.5 times a month [29,34] and 16 times a month [31]. Ross [34] reports that the use declined and leveled off from 1.5 times per month in the beginning to 0.4 times per month after 12 months. In two studies, the percentage of the sample that used the intervention was reported as 76% (32/43) [29] and 80% (43/54) [34]. The pages in the EHR that were the most reviewed by patients were the clinical notes and the lab results [29,34].

The accessing of the intervention was also reported by Berman [26], who found that patients accessed their intervention 16 times a month. Lin reported that the percentage of the sample accessing the intervention was 31% (95/305) [35], and Nguyen reported that 75% of patients (28/37) accessed the intervention [33].

The use of electronic messaging was reported on in six studies [26,29,31,34-36]. Three were publications on sharing EHR [29,31,34], two were on self-care techniques [35,36], and one was on monitoring disease/symptoms [37].

A large percentage of subjects used electronic messaging. Ralston [29] found that 100% of patients (39/39) used email, Ross [34] found 76% (41/54) did, and Cruz [26] found 90% (19/21). The intensity of messaging use varied per patient per year from 1.2 [34], to 5.9 [37], to 8.7 [35], and to 55.2 [31] times. In the latter study, electronic reminders were sent. In one study, it was reported that 73% of messages (302/414) were sent outside of office hours [35].

In three studies, the content of the asynchronous communication [31,34,35] was reported. Kwon [31] reported that the main topics were nutrition, diabetic complications, exercise, and other aspects of diabetes management. Lin described that the main topic of 42% of messages (32/76) was biomedical concerns, and 14 messages were “for your information” (18%), and the latter type of message was significantly more common in electronic messaging than in telephone messaging [34]. Urgent messages were conveyed by telephone [35]. Ross [34] reported that electronic messages appear to supplement telephone messages. The main topics in electronic messaging were scheduling appointments, getting medication refills, asking questions about medication, getting test results, reporting “feeling ill”, and getting assistance to interpret test results.

The usability of electronic messaging was shown for a variety of experiences, and overall, patients were positive about using electronic messaging. In one study, patients found the intervention helpful, easy to navigate, and would recommend it to others [27]. In another study, patients felt that the doctor understood their problems better and explained the information better when using email [34]. Patients prefer email as a way to send information and psychosocial messages to their doctor. In one sample, 75% of patients (132/175) thought they would use this in the future, and 85% (149/175) preferred email to telephone messaging [35]. Additionally, 162 out of 341 (48%) were willing to pay for online correspondence with their physician. Of those willing to pay, the median amount cited was US $2 per message [35]. Cruz found that patients preferred using the Internet rather than paper when monitoring their health [26]. Health care utilization through a patient portal led to higher patient satisfaction [35]. Patients seemed to appreciate the fact that they could communicate with the clinic and conduct administrative actions asynchronously instead of using the telephone. In one study, the content of the communication was studied, revealing that patients had specific questions about medication and tests but also wanted to communicate about “feeling ill” [34]. Patients seemed able to estimate correctly when to use the portal or when to use the telephone for messages, as the telephone was used for urgent messages [35].

Problems concerning usability can be expected when using technology, and these problems might influence usage. Minor usability problems were described in three studies in this review [26,27,39], but none had a large effect on the use.

### Does It Help? Effects on Health Behavior

Multimedia Appendix 2 shows that of the 15 studies aimed at improving health behavior with Internet-based interventions including asynchronous communication, seven reported results on health behavior. The outcomes were in three different areas of health behavior: knowledge [38], health care utilization [35-37,40], and self-efficacy/self-management [27,33,34,36].

Increased knowledge was realized in a study involving rural women with a chronic disease who lived a long distance from the clinic [38].

Health care utilization in the form of visits to physicians did not decrease significantly, but a decrease was shown in the case of back pain patients (*P*=.07) [36] and asthma patients (*P*=.07) [37], although not statistically significant.

In the area of Internet-based support of self-efficacy/self-management, several results were reported. In the case of pain management, Internet-based interventions seemed to increase patients’ self-efficacy in using non-medical techniques [27], self-care orientation in back pain [36], and managing dyspnea [33]. In patients with congestive heart failure, the general adherence to therapy increased when patients shared their EHR with their providers and communicated asynchronously about the content and implications of the EHR [34].

### Does It Help? Effects on Health Outcomes

Health outcomes are important indicators for providers to guide the therapy of chronically ill patients. Health outcomes as a result of using an intervention with asynchronous communication were described as clinical and physical symptoms, psychosocial outcomes, and satisfaction. They were reported in 12 studies [9,27-33,35-37,39,41].

Outcomes for clinical symptoms were shown in four studies of diabetic patients. Improvements were shown in HbA1c level [9,29,31,32], weight, cholesterol, high-density lipoproteins [9], and blood pressure [32]. In a study of asthma patients, the forced expiratory volume (FEV) and the control of asthma were shown to increase with Internet-based support [37]. These were positive results for Internet-based interventions with asynchronous communication, but only McMahon looked at specific aspects of the intervention. He found that improved health outcomes were related to the frequency of use of the intervention [32].

Improved physical symptoms were also observed when using Internet-based self-care techniques. Berman and Lorig demonstrated a decrease in back pain in patients [27,36], and Ghahari demonstrated a decrease in fatigue-impact for patients with multiple sclerosis [28]. Nguyen reported increased arm endurance with exercise [33]. Berman discussed a relationship with a specific aspect of the intervention: logging on to the intervention seemed to decrease the patients’ pain immediately [27].

Varied psychosocial outcomes were shown in the studies of interventions with asynchronous communication for chronically ill patients. In one study, personal well-being increased [28] for both intervention groups (interactive and information only) in comparison to the control group with usual care. Meer showed improvement in quality of life for asthma patients [37]. An increased acceptance of the illness was also shown [39], as well as increased self-esteem [30,39], empowerment, and social support [30]. Weinert described a decrease in stress, depression, and loneliness [39]. Lorig found that patients felt less disabled, whereas role functioning improved and health-related distress decreased [36]. Patients seemed to feel better when they had an Internet-based connection with their providers.

### Does It Help? Effects on Patient Satisfaction

Satisfaction with the overall care from the clinic increased in one study when patients used the Internet-based connection with their provider via the patient portal [35].

## Discussion

### Results of Search

The literature search revealed that there are few studies of the effects of asynchronous communication on self-management of chronically ill patients. Only Lin had an Internet-based patient-provider communication system as the focus of an RCT [35]. The literature search yielded another 14 studies in which asynchronous communication was described as part of the intervention. The RCTs were mainly performed in the United States with patients with specific chronic conditions. To glean information about the introduction of asynchronous communication for tailor-made health care, more evidence from other countries and patients with diverse chronic conditions is required.

### Quality Appraisal

When reviewing the methodological quality of the studies, a certain amount of lack of blinding was noted. This lack of blinding is inevitable for technical reasons when the use and effect of digital communication on health behavior and health outcomes is being examined. In assessing the performance bias in RCTs using these techniques, it can be argued that not too much weight should be given to this aspect of quality appraisal.

### Results on Use and Usability

Although 12 studies report on the frequency of use of the intervention, none specifically examined why and when patients log on to the intervention. The results of this review suggest that it might be interesting to find out more about the meaning of the frequency of use by patients. Comparisons with other publications about the frequency of access of Internet and communication technology by patients show varied results from increasing use [42] to declining use [34,43], but these results did not differ from those for usual care [44]. Kwon shows that the frequency of use increases when reminders are sent by the provider [31], resulting in increased health outcomes. Ross [34] showed a decline in use but an increase in adherence. Possibly the effect is not in the actual use but in having the connection to the provider, who can be contacted if necessary. It is also possible that the patients’ questions have been answered and they know what to do.

In the studies where email use was measured, a high percentage of patients (>75%) used it [29,34]. Patients seem to be interested, but it is not yet clear when they feel the need to use it or whether being connected is enough to feel satisfied and more in control of their health. Perhaps patients experience the connection as a supporting factor in their self-management. The virtual presence of the professional through the digital connection might have effects that could be interesting. Perhaps patients do not need to “check” the digital connection by using it after a time, as they feel confident knowing the digital connection with the provider can be made whenever they need it. The provider is always present and can be approached if the need arises. In research on social support, it is shown that merely the availability of support is helpful and related to higher levels of well-being [45,46]. More understanding of the effects on patients of asynchronous communication could lead to increasingly tailor-made health care.

From the viewpoint of transition to integration of modern communication technologies, several threats to successful integration can be identified. It is certainly a threat if patients are not able to distinguish issues that are acute or (life)threatening versus non-acute, but we found no evidence to support this. We found that patients know when to use the telephone, because urgent messages were conveyed by the telephone [35]. They also know when to use asynchronous communication; patients preferred sending non-urgent messages such as “for your information/feeling ill” by email [34,35]. In addition, informational and psychosocial messages are sent via email as opposed to the telephone [35].

Also with regard to integration of ICT, it is interesting that patients are prepared to pay for email service with their provider [35]. The fact that 73% of messages (302/414) are sent outside of office hours suggests that time and place might be a factor [35].

The content of the electronic messages suggests that patients are willing to participate actively because they share more information than strictly necessary. They have a variety of issues they want to communicate about when using asynchronous communication. Understanding what these issues are is important for further implementation. In addition to “for your information/feeling ill” messages, they have health issues that they want to clarify, such as biomedical and medication concerns and receiving test results and assistance to interpret them. What seems to be happening is that asynchronous communication is used to communicate information that may or may not be relevant, but it satisfies patients to send it. They have taken action by sending the information, and it is now up to the health care provider to say whether it is relevant and if action is needed. This is the start of “shared-decision making”. With asynchronous communication, patients seem to make use of the option to share their worries and their psychosocial condition with their provider. This is an indication for willingness for further patient participation.

Age does not seem to be a factor in the use of asynchronous communication, given the advanced age of the participants. This is relevant because most patients with a chronic disease belong to the older segment of the population [1]. In the literature, older age has been identified as a barrier to the use of Internet communication technology [47-49]. It can of course be argued that patients who are included in the sample must have Internet access and minimum competencies to use it. It does show, however, that age is not an unsurpassable barrier. It may even be so that the benefits of having a virtual connection with the provider stimulates patients to use the Internet connection or at least understand how to use it, no matter what their age.

### Results Relating to Health Behavior

In the seven studies where health behavior is described, improvements are shown when using the interventions [27,33-38]. The Internet-based intervention is therefore an option in regard to providing support self-management at a distance.

The meaning to the patient of the digital connection to the provider is interesting. Does having the connection at your fingertips give a reassuring feeling? Does sitting down and logging on to the connection feel like the first step in self-management and being assertive about your needs? Some results on health behavior may point in this direction. The two studies that used an intense (with an online coach) and a less intense intervention (without an online coach) show that a less intense intervention is just as effective [28,38]. This again raises the question of whether the connection alone is enough to improve health behavior, or whether a more complex intervention is necessary to gain an effect. In another study, it is suggested that logging on has an immediate impact on pain reduction [27]. This stresses the need to clarify which aspects of eHealth interventions are effective for patients. Is it the direct connection via Internet with the provider, is it the online coach, or is it the tailored information? The latter may be a very interesting point, as this interactive communication makes it possible to obtain detailed information about health management from the provider and the patient. In all other settings, such as in the consulting room, using the telephone or writing letters, it requires more effort to obtain the information necessary for tailor-made intervention.

Two studies found a trend to a significant decrease of health care utilization in the form of visits to physicians when using an Internet-based intervention [36,37]. It may be that asynchronous communication plays a role in this change in health behavior because patients can discuss their health concerns interactively with their provider. The triggers for this health behavior might be less time spent travelling to the physician and in the waiting room; the convenience and fact that no travel is required make the interaction more economical. However, this aspect has not been studied.

The content of the communication in the study by Ross shows that patients have precise questions about medication and tests, but that they also want to communicate about “feeling ill”. The results show that general adherence increases. However, the question of whether asynchronous communication about these issues affects the general adherence is not raised [34]. There may be some support for this conclusion in the Lin study, where patient satisfaction increased when they were able to communicate “for your information” messages through electronic messaging [35].

### Results on Health Outcomes

Improved health outcomes were shown in 11 studies using Internet-based interventions, including electronic messaging. In studies where the intervention was complementary to usual care, an improvement could be expected. In studies with an intervention as a substitute for usual care, similar outcomes from intervention and usual care can be seen as a positive result. However, improvements were also reported in studies in which the intervention was a substitute, thus showing that the Internet intervention has better results than face-to-face care. This calls for more testing of these interventions as a substitute for usual care in larger samples of diverse patients.

### Limitations

The Internet-based interventions in the studies consisted of different components, such as peer-support groups, sharing medical records, self-management programs, and patient portals. The component they all had in common was electronic messaging. The multicomponent aspect of the studies made it difficult to trace the exclusive effect of the intervention to the asynchronous communication.

Electronic messaging was not evaluated separately in these studies. It can be concluded that the effect of asynchronous communication is not adequately shown in these studies and that many questions can be raised about the precise effect of Internet-based asynchronous communication between patients with a chronic condition and their providers. This is a limitation. However, there were many positive findings about electronic messaging in relation to telephone messages and other forms of messaging.

### Future Research

Research is needed to determine the technical characteristics of effective asynchronous communication with patients for specific disease categories where specific health behavior is needed by specific patients. The meaning of the virtual connection with the provider should be explored. This may be a very basic intervention with a large effect.

Additional testing is needed to clarify what patients want to discuss with their providers and how shared decision making about these issues can be effective.

The effects of asynchronous communication on self-management for larger samples of diverse patients with a chronic condition require examination. A clear definition of desirable outcomes is needed. The desired results for health behavior should be operationalized with regard to “the ability to adapt and self-manage in the face of social, physical, and emotional challenges” [5]. In this review, three categories of health behavior could be distinguished as outcomes: knowledge, health care utilization, and self-management/self-efficacy.

We also advise further testing of Internet interventions as a substitute for usual care because significant health outcomes were found in this review.

### Conclusions

It can be concluded that using asynchronous communication in health care may be an important instrument to increase patient participation leading to self-management. After reviewing the literature, the answer to both research questions seems to be positive: asynchronous communication is used by patients and it helps to increase the effects on health behavior and health outcomes, at least for some. Patients seem to be interested in using email and understand how to use it. They use email for questions about biomedical concerns, medication, and test results, as well as to inform the providers about non-urgent health issues. They tend to prefer email to telephone for this communication. They also understand when they can use email or when contact by telephone is needed.

From the viewpoint of the new definition of health with an emphasis on self-management and patient participation, it seems possible to take steps towards sustainable health care by implementing asynchronous communication, as it enables patients to communicate effectively about their perceived health problems and their adaptation to health problems.

## Multimedia Appendix 1

Methodological quality randomized clinical trials.

## Multimedia Appendix 2

Results reported on use of the intervention, health behavior, and health outcomes.

## Abbreviations

EHR: electronic health record
FEV: forced expiratory volume
ICT: information and communications technology
NICE: National Institute for Health and Care Excellence
RCT: randomized controlled trial
WHO: World Health Organization
