# Supplementary material for: The Effects on Health Behavior and Health Outcomes of Internet-Based Asynchronous Communication Between Health Providers and Patients With a Chronic Condition: A Systematic Review
Source: J Med Internet Res. 2014 Jan 16;16(1):e19. doi: 10.2196/jmir.3000 (PMC3913926; doi:10.2196/jmir.3000)
Supplement: Supplementary file 1 [file jmir_v16i1e19_app1.docm]

### ****Multimedia Appendix 1: Methodological quality randomised clinical trials****

|  | **Bias** | **Selection** | | | **risk** | **dir.** | **Performance** | | | **risk** | **dir.** | **Attrition** | | | **risk** | **dir.** | **Detection** | | | | | **risk** | **dir.** | **Total score** |
| --- | --- | --- | --- | --- | --- | --- | --- | --- | --- | --- | --- | --- | --- | --- | --- | --- | --- | --- | --- | --- | --- | --- | --- | --- |
|  | Author | A1 | A2 | A3 |  |  | B1 | B2 | B3 |  |  | C1 | C2 | C3 |  |  | D1 | D2 | D3 | D4 | D5 |  |  |  |
|  |  |  |  |  |  |  |  |  |  |  |  |  |  |  |  |  |  |  |  |  |  |  |  |  |
|  | Berman [27] | y | y | y | l |  | n | np | np | ? |  | y | y | y | l |  | n | ± | ± | np | np | h | 0 | ± |
|  | Bond [9] | y | y | y | l |  | y | np | y | l |  | y | y | y | l |  | y | y | y | y | y | l |  | l |
|  | Cruz [26] | y | y | y | l |  | y | np | y | l |  | y | y | y | l |  | n | y | ? | np | np | h | ? | l- |
|  | Ghahari [28] | y | y | y | l |  | y | ± | y | l |  | y | y | ± | l |  | n | y | y | y | y | ? |  | l |
|  | Hill [30] | ? | ? | ? | h | 0 | y | np | ? | ? |  | y | y | y | l |  | y | ± | y | y | y | l |  | ± |
|  | Kwon [31] | y | y | y | l |  | y | np | n | l |  | y | y | y | l |  | ± | y | y | n | n | l |  | l |
|  | Lin [35] | y | y | y | l |  | y | np | np | l |  | y | y | y | l |  | y | y | ± | np | np | ? | 0 | l |
|  | Lorig [36] | ? | ? | y | ± | 0 | y | np | ? | l |  | y | y | y | l |  | y | y | y | ? | ? | l |  | l |
|  | McMahon [32] | y | y | y | l |  | y | np | ? | l |  | y | y | y | l |  | y | y | y | n | n | l |  | l |
|  | Meer[37] | y | y | y | l |  | y | np | n | l |  | y | y | y | l |  | y | y | y | np | y | l |  | l |
|  | Nguyen [33] | Y | ? | y | l |  | Y | np | np | l |  | y | y | y | l |  | y | y | y | np | ? | l |  | l |
|  | Ralston [29] | y | y | y | l |  | y | np | n | l |  | y | y | y | l |  | y | y | y | np | np | l |  | l |
|  | Ross [34] | y | y | y | l |  | y | np | ± | l |  | y | y | y | l |  | y | y | y | np | np | l |  | l |
|  | Weinert [38] | y | y | ? | ? | ? | y | ± | ? | l |  | y | ? | y | ? | 0 | y | y | n | np | np | h | ? | ± |
|  | Weinert [39] | y | y | y | l |  | y | np | np | l |  | y | y | y | l |  | y | ± | ? | np | ? | l |  | l |

Y=yes, N=no, ±=more or less, na=not applicable, np=not possible, ?= unclear, l=low risk of bias, h= high risk of bias, 0= no effect expected of the increased risk of bias.,. In each category (A1-3, B1-3, C1-3, D1-5) questions are asked to determine the risk of bias.

Risk: If all questions in one category are answered with a yes the risk of that type of bias is low. If this is not the case the risk has been analyzed by two researchers separately and the outcomes were discussed so that a decision could be made.

Dir.: The likely direction of effect of the bias is assessed

Total score: If all categories score low risk of bias, the total score is low. When three categories score low risk of bias and one category scores a high risk, the total score is low-. If two categories score low risk of bias and the other two are unknown or have a high risk with a 0 direction, the total score is ±.

**Selection bias**: systematic differences between the comparison groups.
A1 An appropriate method of randomization was used to allocate participants to treatment A2 There was adequate concealment of allocation A3 The groups were comparable at baseline, including all major confounding and prognostic factors

**Performance bias**: systematic difference between groups in the care provided, apart from the intervention under investigation.
B1 The comparison groups received the same care apart from the intervention(s) studied B2 Participants receiving care were kept ‘blind’ to treatment allocation B3 Individuals administering care were kept ‘blind’ to treatment allocation

**Attrition bias**: systematic differences between the comparison groups with respect to loss of participants.
C1 All groups were followed up for an equal length of time C2 The groups were comparable for treatment completion C3 The groups were comparable with respect to the availability of outcome data.

**Detection bias**: bias in how outcomes are ascertained, diagnosed or verified.
D1 The study had an appropriate length of follow-up D2 The study used a precise definition of outcome D3 A valid and reliable method was used to determine the outcome D4 Investigator were kept ‘blind’ to participants’ exposure to the intervention D5 Investigators were kept ‘blind’ to other important confounding and prognostic factors
