# Supplementary material for: The Effects on Health Behavior and Health Outcomes of Internet-Based Asynchronous Communication Between Health Providers and Patients With a Chronic Condition: A Systematic Review
Source: J Med Internet Res. 2014 Jan 16;16(1):e19. doi: 10.2196/jmir.3000 (PMC3913926; doi:10.2196/jmir.3000)
Supplement: Supplementary file 2 [file jmir_v16i1e19_app2.pdf]

Multimedia Appendix 2. Results reported on use of the intervention, health behaviour and health outcomes.

Primary author	Outcomes usage digital intervention/ asynchronous communication<br><br>A actual use<br>B asynchronous communication<br>C usability	Outcomes health behaviour<br><br>A knowledge<br>B healthcare utilization<br>C self efficacy/self-management	Outcomes health<br><br>A clinical<br>B physical<br>C psychosocial	Patient satisfaction
Berman 2009	A median use over 6 wks 22.5 times. One visited 211 times,<br>A 78% had intention to do exercise at log off. In follow-up survey 95.3% said they did the exercises without going on line<br><br>B not reported<br>C 81% believed intervention to be helpful<br>C 88.4% easy to use<br>C 95.3% would recommend intervention to others<br>C all modules were perceived as helpful<br>C 10 had limited difficulties using site: log on and navigating or downloading<br>C 2 people did not appreciate the intervention<br>C "having the intervention handy confirmed that someone was anxious about their pain"<br>C "felt like you were talking to me"<br>C made them more aware of their pain experience and made them focus on self care<br>C suggestions to add more content, additional introductory text, changing layout, communication with other users	P= 0.05<br>C Awareness of responses to pain +<br>C Confidence using non-medical techniques +	P= 0.05<br>B Pain intensity +<br>B Pain scores at log on/off suggests immediate impact on reduction of pain	
Bond 2007	B not reported		P = 0.001<br>A HbA1c p= .01<br>A Weight p= .001<br>A Cholesterol p= .05<br>A HDL p= .05	
Cruz 2007	A minor technical problems<br>9 with internet connection<br>5 with PIKO<br>5 with paper diaries<br>A Patients tried both diaries. Internet could only be entered			

	daily, whereas paper more days could be entered at a time<br>A looking at previous data was considered easier on internet<br><br>B not analyzed<br><br>C Preference for internet diaries<br>C Internet diaries less complete<br>C most important features of intervention according to patient:<br>- asthma diary 93%<br>- educational content 82%<br>- receiving information about asthma 85%<br>- exchanging messages with doctor 85%<br>- self-assessment tool before consultation 79%<br>- receiving messages about medication 69%<br>- receiving messages about consultation 67%<br>- 50% were prepared to monitor always<br>- 33 % prepared to monitor daily, rest less			
Ghahari 2010	A no sign diff participation rate (nr of sessions completed) I A / I B<br><br>B not reported		B Physical subscale Fatigue Impact p=.04<br><br>C Personal Wellbeing (for I B) p=.03	
Hill 2006	B not reported		C Self-esteem p= .016<br>C Social support p= .038<br>C Empowerment p= .016	
Kwon 2004	A average frequency monitoring 71.5±36.2 (I) and 38.1±24.8 (C)<br>A mean Logon 42.3±32.2/patient<br>A have more contact with physician.<br>A received medical advice according to recent data<br><br>B Total number of e-mail questions was 167.<br>Mean number of questions posted per patient in 12 weeks was 13.5 ± 14.7.<br>Detailed list of questions asked via the Internet		A I HbA1c p= .001	

	homepage by the patients in the intervention group to their health care providers			
Lin 2005	A 85% obtained user account for portal<br>A 31% used the portal<br>A 175 admin. requests and 239 clinical messages (phone and e-mail) sent by portal patients (1 message/day for every 250 patients)<br>A 27% sent during and 73% outside clinic hours.<br><br>B n=76 asynchronous messages<br>Urgent message 3% (of n)<br>Medication question 1%<br>Medication action 21%<br>Biomedical concerns/questions 42%<br>Test results 17<br>Test action 7<br>Psychosocial concerns 9%<br>“for your information” 18%<br>Home monitoring 5%<br>Prevention 5%<br>Miscellaneous 18%<br><br>C Patients more likely to send informational and psychosocial messages by portal than by phone<br>C 75% likely to use portal in future<br>C 85% prefer portal to phone<br>C Of all surveyed patients, 162/341 (48%) were willing to pay for online correspondence with their physician. Of those willing to pay, the median amount cited was US $2 a message<br>C 81% of users said it saved them a phone call<br>C 33% said it saved them a visit<br>C weak positive correlation between frequent users and<br>- satisfaction with portal services p=.02<br>- improved comm. with clinic p= .01<br>- satisfaction with physician messaging p=.03	B improved communication with clinic p= .001		Patient satisfaction overall care p= .04
Lorig 2002	A 69% sent 1 or more emails to the group.<br>A Active members posted a mean of 8 messages in a year.	B Health care utilization decreased but ns p= .07<br><br>C self care orientation	B Pain p= .045<br>C Disability p= .02<br>C Role function p= . 007<br>C Health distress p= .	

	B not reported<br><br>C 41% read most of the email messages, 37% read only emails with subjects of interest.	p= .002<br>C self efficacy p= .02	001	
McMahon 2005	A Frequent intervention use was related to improved health outcomes<br>A greater amount of uploads of monitoring data related to improved health outcomes p= .02<br><br>B not reported		A HbA1c p= .05<br>A persistent users greater improvement HbA1c than intermittent p= .05 or education and usual care p= .05<br>A Blood pressure p= .01<br>A cholesterol p= .05	
Meer 2009	A 5.9 contacts/pat asthma nurse (1yr)<br>A Report lung function scores 108 days<br><br>B not reported	B physician visits p= .07	A FEV p= .025<br>A Asthma control p= .001<br><br>C Asthma QOL p= .001	
Nguyen 2012	A Logins median 148 over 12 mths (n= 43)<br>A 75% used website at least once<br><br>B not reported<br><br>C preferred using website to smart phone for recording daily exercises and symptoms	B high levels support perceived<br><br>C arm endurance p= .04<br>C self efficacy trend p= .06	A Severity of dyspnoea no difference	
Ralston 2009	A 76% accessed EHR (n=39)<br>A 43% HbA1c upload<br>A 33% entered other data<br>A Uploads of HbA1c levels show trend to improvement<br>A 1146 web pages EHR viewed in 12 mths (=2.3/mth)<br>- 26% transcribed notes<br>- 20% labresults<br>- 9% problem lists<br>A number of page views is not related to HbA1c improvement<br>A uploads of blood glucose levels (n=189) trend to improvement p= .09<br><br>B 69% used Email		A HbA1c p= .01	
Ross 2004	A Logins 581 by 43 pat. (n=54)<br>A 0.4 hitdays/pat/mth<br>A Clinical notes and lab results viewed 95%<br>A Educ.guide reviewed 63%<br>A Use high first 3 mths<br><br>B 24% use e-mail	C Adherence general p= .01		

	B e-Mail volume 63 by 95% patients<br><br>C Doctor-patient comm. satisfaction trend			
Weinert 2008	B not reported	A Health knowledge (A+B gained sign.) p= .000		
Weinert 2011	A 4 had problems with using computer<br>2 had problems with internet<br><br>B not reported		C Stress p= .005<br>C Depression p= .01<br>C Self-esteem p= .018<br>C Acceptance of illness p= .001<br>C Loneliness p= .04	
